# Ethnomedicinal Plants Used by Traditional Healers to Treat Oral Health Problems in Cameroon

**DOI:** 10.1155/2015/649832

**Published:** 2015-10-01

**Authors:** Michael Ashu Agbor, Sudeshni Naidoo

**Affiliations:** Department of Community Dentistry, University of the Western Cape, Private Bag X1, Tygerberg, Cape Town 7505, South Africa

## Abstract

*Objectives.* The objective of the study was to determine the therapeutic methods used by traditional healers to treat oral diseases in Cameroon.* Methods.* A total of 200 traditional healers with a mean age of 50.4 ± 14.2 years from all the provinces of Cameroon were studied using questionnaires. Information elicited was the local names of the medicinal plants used for the management of oral problems, their routes of administration, and methods of usage. Identification of live or dried plants or photographs of sample of the plants was done by a taxonomist.* Results.* The majority of the participants were males urban dwellers aged 41–50 years, 112 (56.0%) practice as herbalists and 56 (28.0%) were trained on medications preservation, 77(56.6%) treat diseases inside or outside the mouth, and 9.0% reported being specialist in oral diseases treatment. Of the 52 plants identified, 48 are used in the management of toothache, sore throat, mouth sores, abscess, broken tooth and jaw, tooth sensitivity, mouth thrush, dental caries, gingivitis, sinusitis, tonsillitis, xerostomia, oral syphilis, oral cancer, TMJ pain, halitosis, and tooth bleaching and 4 plants are used for dental extraction. Roots, leaves, and bark were the parts of plants used and some minerals as adjuncts.* Conclusion.* The study provides comprehensive information on therapeutic methods employed by traditional healers for the treatment of oral diseases.

## 1. Background

Plants produce chemicals as primary and secondary metabolites which have beneficial long-term health effect and also are used effectively to treat diseases. Specifically, it is the secondary metabolites that exert therapeutic actions in humans. It has been stated that more than 30% of the entire plant species, at one time or another, are used for medicinal purposes necessarily due to the amount and type of secondary metabolite they contain [1]. These drugs of plant origin have saved lives of many residents of developing countries because of their good values in treating many infectious and noninfectious chronic diseases.

In spite of the overwhelming influences and the dependence on modern medicine and tremendous advances in synthetic drugs, large segments of the world population depend on drugs from plants [1]. Population rise, inadequate supply of drugs, prohibitive cost of treatments, side effects of several allopathic drugs, and development of resistance to currently used drugs for infectious diseases have led to increased emphasis on the use of plants as sources of medicines for a wide variety of human ailments [2–5]. According to World Health Organization (WHO), over three-quarters of the world population rely on plants and their extracts for healthcare needs [6].

Poverty, inadequacy of health services, shortage of health workers, and rampant shortage of drugs and equipment in existing health facilities make traditional medicine an important component of healthcare in Africa. Traditional healers were reported as first choice healthcare providers when they faced health problems in Ethiopia due to their efficacy and dissatisfaction with modern medicine [7]. In many of the developing countries, the use of plant drugs is increasing because modern life saving drugs are beyond the reach of their countries although they spend 40–50% of their total wealth on drugs and healthcare. As a part of the strategy to reduce the financial burden on the developing countries, it is obvious that an increased use of plant drugs will be followed in the future [1, 2].

The global need for alternative prevention and treatment options and products for oral diseases that are safe, effective, and economical comes from the rise in disease incidence particularly in developing countries, increased resistance by pathogenic bacteria to currently used antibiotics and chemotherapeutics, opportunistic infections in immune-compromised individuals, and financial considerations in developing countries [4, 5]. For example, bacterial resistance to most (if not all) of the antibiotics commonly used to treat oral infections (penicillin and cephalosporins, erythromycin, tetracycline and derivatives, and metronidazole) has been documented [8]. These drugs also alter oral microbiota and have undesirable side effects such as vomiting, diarrhea, and tooth staining [3]. The herbal products today symbolize safety in contrast to the synthetics that are regarded as unsafe to human and environment.

Traditional Chinese medicines have been used to treat some of these orofacial problems for more than 2000 years [9]. Zheng and colleagues reported that Chinese traditional medicines are effective in treating oral diseases including recurrent aphthous stomatitis, oral lichen planus, leukoplakia, and Sjögren's syndrome but remarked that most of them lacked standard criteria of posttreatment assessment and laboratory evidence [9]. It has also been reported that traditional Chinese medicine and naturopathic medicine resulted in significantly greater reduction of pain and psychosocial interference in temporomandibular disorders than the state-of-the-art specialty care [10].

Hollist (2004) [11] reported that about 10 different oral/dental conditions are treatable with plants in traditional health practice, namely, toothache/decay, gingivitis, ulcerative gingivitis, angular stomatitis, mouth ulcers, swollen tonsil, oral thrush, tonsillitis, and black tongue. The result of a study in the Tanga Region of Tanzania showed that dental patients are commonly treated by traditional healers and more than half of inhabitants with toothache sought treatment from traditional healers, where they had all been treated with local herbs. This health seeking behavioral pattern was not altered by the establishment of modern emergency oral care in rural health centers and dispensaries did not influence the villagers' use of the traditional healers [12]. More than half of the Malaysian aborigines (56.8%) used traditional medicine for relief of orofacial pain [13].

There is paucity of literature in traditional therapeutics in the treatment of orofacial diseases in Cameroon. Few studies in Cameroon showed that native herbs are common self-medicament for oral diseases and that traditional healers are involved in tooth extractions [14] and the treatment of other oral diseases like the oral manifestations of HIV/AIDS [15]. It was also documented that Cameroonians use herbs for self-medication for oral health problems [16]. Hence, the objective of this study was to determine the therapeutic methods used by traditional healers to treat oral diseases.

## 2. Methods

### 2.1. Study Design

This study set to determine the therapeutic methods used by traditional healers to treat oral diseases was conducted as a cross-sectional study.

### 2.2. Study Setting

This study was conducted in all the 10 regions of Cameroon. This is a central African country with a population of 21.7 million, without dental school, and has about 220 practicing dentists revealing a low dentist population ratio of about 1 : 10,000.

### 2.3. Participants

This study included all forms of traditional healers that belong to and participate in the regional activities of their association.

### 2.4. Instrument

A self-administered questionnaire was used to elicit information from traditional healers. Those traditional healers who were unable to read or write were interviewed and their responses captured. Information elicited was demography of the traditional healers, the local names of the medicinal plants/products used for the management of orofacial problems, and their routes of administration and methods of usage.

### 2.5. Procedure

Traditional healers assembled by their regional head in the monthly meetings were contacted and the purpose of the research was explained to them. Those that consented were recruited and studied. They were administered the questionnaire. Plants samples were requested from traditional healers who could present life plants. The plant samples and products were collected from the traditional healers either fresh or in dried forms and photographs depicting the habit of plants that could not be harvested were taken. These plants samples were subsequently taken to the Department of Botany at the University of Dschang, Cameroon, for identification by a taxonomist.

### 2.6. Ethical Concerns

Authorization to conduct this research on traditional healers in all the regions of Cameroon was obtained from the Ministry of Higher Education and Scientific Research of Cameroon and the Senate Research Ethics Committee of the University of the Western Cape (UWC), South Africa. Written informed consent was obtained from traditional healers who agreed to participate in the study.

### 2.7. Data Analysis

Data obtained were analyzed using the Statistical Package for Social Sciences (SPSS version 17.0, SPSS Inc., Chicago, IL, USA) and summarized using descriptive statistics and presented as frequencies and percentages.

## 3. Results

A total of 200 traditional healers with a mean age of 50.4 ± 14.2 years from all the provinces (regions) of Cameroon participated in this study. The majority 48 (24.0%) were aged 41–50 years. Males 138 (69.0%) were more than females 62 (31.0%). More than a third 64 (32.0%) had a primary school education, 57 (28.5%) secondary education, 47 (23.5%) a tertiary education, and 32 (16.0%) only informal education. A total of 110 (55.0%) of the participants resided in urban areas. More than half 112 (56.0%) of the traditional healers practice as herbalist, as diviners 28 (14.0%) and both herbalism and divination 60 (30.0%). Fifty-six (28%) of the traditional healers were trained on how to preserve their medications. Seventy-seven (56.6%) participants indicated treating disease inside or outside the mouth but only 9.0% reported being specialist in treating oral diseases only (Table 1). The mean number of patients seen in a week was 13 ± 8 with majority of them 49 (25.0%) treating 13–16 patients weekly (Table 2).

A total of 52 plants were identified, 32 of which are used in the management of oral problems in form of toothache, sore throat, mouth sores, mouth ulcers, bullous lesion, abscess, broken tooth, dentine sensitivity, mouth thrush, dental caries, gingivitis, sinusitis, tonsillitis, dry mouth, oral syphilis, and oral cancer (Table 3), 16 used for specific oral diseases (Table 4), and the remaining four used for dental extraction (Table 5). Roots, leaves, and bark were most common parts of identified plants used for treatment of oral problems (Table 6). The minerals used as adjunct were calcium carbonate, alum, bicarbonate solution, and white and yellow sulphur (Table 7).

## 4. Discussion

Medicinal plants play an essential role in primary healthcare as they are used to treat wide varieties of oral diseases because they possess antibiotic and anti-inflammatory properties. However, the main drawbacks of tradition health practices endogenous uses include incorrect diagnosis, imprecise dosage, low hygiene standards, the secrecy of some healing methods, and the absence of written records about the patients [17]. There is a common perception that traditional healers do not have any equipment for the diagnoses of oral pain or evaluate posttreatment pain assessment thereby depending on the signs and symptoms. However, Ndenecho had observed that the diagnosis of disease by traditional healers is not limited to direct observation and tests. Divination was also used as 44.0% of the participants practiced it. Preservation and storage of medicinal plants products is a major problem facing traditional medicine in many developing countries like Cameroon. In this study, only 56 (28.0%) of the traditional healers were trained on medication preservation. This invariable means that the postharvest storage and processing technologies are poor and need to be developed to produce the value added to finished products that may be directly utilized by the industry. The establishment of training to facilitate the production of good quality, safe, and efficacious therapeutic is therefore necessary.

The secondary metabolites of plants possess medicinal properties and these medicinal qualities of plants have influenced their price for centuries [1]. This study documented 52 plants used in the management of various forms of oral problems and dental extractions. The majority of the identified plants were used in the management of oral pain mainly toothache. This may not be unconnected with the fact that attention seeking for toothache is very high; thereby every care provider equips their arsenal with best and effective treatment option. Laboratory analyses of several medicinal plants have been showing that they have antimicrobial and anti-inflammatory components as primary or secondary metabolites. This study confirms why most of the oral diseases treated were related to pain and infections.

The resolution of toothache from pulpitis occurs because many medicinal plants cause pulp necrosis and mummification of the pulp tissue. However, this resolution is temporary because further dental caries leads to reinfection of dental pulp tissues and consequent reoccurrence of pain. The 10 different oral conditions (toothache/decay, gingivitis, ulcerative gingivitis, angular stomatitis, mouth ulcers, swollen tonsil, oral thrush, tonsillitis, and black tongue) treatable with plants in traditional health practice [11] documented in the literature were also noted in this study except for black tongue. However, other additional uses like oral syphilis, oral cancer, tooth bleaching, halitosis, and dental extraction reported in this study reflect the comprehensive and nationwide spread of the study.

Other studies in Cameroon have indicated that, apart from their specific uses for dental treatments, some of these plants are used for other medical treatments and as food as well [18]. Most of the plants in this study are used as food* or* as food additives and they include* Canarium schweinfurthii, Vernonia amygdalina, Anacardium occidentale, Cocos nucifera, Allium sativum, Citrus sinensis, Carica papaya, Allium cepa, Ricinodendron heudelotii, Mangifera indica, Zea maize,* and* Syzygium aromaticum*. This is in line with other studies carried out by Dibong et al. [19] and Din et al. [20] who found out that some of these plants and their parts such as roots, rhizomes, tubers, leaves, stem, wood, bark, flowers, seeds, and fruits are used in various purposes in their daily life. Cloves (*Syzygium aromaticum*), known locally in Cameroon as “clue de geof” because of their nail-like presentation, are a common food spice that has natural antiviral, antimicrobial, antiseptic, and antifungal agent with aphrodisiac and circulation-stimulating capacities [21, 22]. Approximately, 72–90% of the essential oil extracted from cloves has eugenol which has a variety of uses in dentistry (pulp dressings, cavity liners, and dry socket dressing) [21]. The clove has also been used in India and China, for over 2,000 years to control both tooth decay and counter bad breath [21]. It has been used extensively in dental care for toothache, sore gums, and oral ulcers relief. Gargling with clove oil can also aid in sore throat conditions and bad breath [22]. In this study, it is used locally to treat toothache by direct application of moist paste to a painful tooth or mouth sore.* Allium sativum* also called garlic is a common food spice and medicinal plant. The paste made from the bulb is used by direct application to a painful tooth to relieve toothache and treat gingivitis. It has broad spectrum antibacterial, antiviral, and fungal activity [23]. Although less effective and cytotoxic to periodontal tissues compared to chlorhexidine, garlic has a high antibacterial effect against human dental plaque microbiota (*Streptococcus mutans, S. sanguis, and S. salivarius; Pseudomembranous aeruginosa and lactobacillus* spp.) even at very low concentrations [24]. The side effect of this plant is chemical burns that are usually associated with mouth ulcers and persistent halitosis from its strong aromatic smell. Deodorization of garlic extract resolves much of this drawback of halitosis. Drinking milk and use of mushroom extract, tea catechins, plant extracts containing polyphenol and phenolic derivatives, and honey have been reported to be effective in suppressing the malodour of garlic [25–29].* Ricinodendron heudelotii* is a plant that produces some oily seeds (Njasang) used for spicing soup. It has been found to have antioxidant and antibacterial properties stronger than most antibiotics [30, 31]. The leaves of* Vernonia amygdalina* (bitter leaf) are used in Cameroon as a green vegetable or as a spice in soup, especially in the popular bitter-leaf soup (ndolé). It is used for calming down toothache by direct application on a cavity. Extracts of the plant have been used in various folk medicines as remedies against helminthic, protozoal, and bacterial infections with scientific support for these claims [32], cancer chemoprevention, and the treatment of diabetes [32].* Ricinodendron heudelotii* is boiled to make hot mouthwash that is used to treat toothache. There is a traditional belief in Cameroon that whatever can be used as food or whatever animals eat without any harm can safely be used for medicine because of its minimal toxicity. The high usage of herbs among the households is an indication of their abundance [33].

The most commonly used plants in Cameroon for the management of dental infections are* Carica papaya, Psidium guajava*, and* Nicotinia tobacum. Other common plants for treatment of oral problems included Spilanthes africana, Eucalyptus saligna, Moringa oleifera, Capsicum frutescens, Ageratum conyzoides, Dichrocephala integrifolia, Persea americana, Ipomoea batatas, Vernonia amygdalina, Garcinia mannii, Garcinia kola*, and* Arnica montana*.

The seed and the pulp of* Carica papaya* had been shown to treat more than 20 diseases and studies have shown that it is bacteriostatic against common oral microorganisms like* Staphylococcus* spp. [34]. The latex of* Carica papaya* has been shown to reduce the growth of* Candida albicans* by 60%; the fruits when used as topical ulcers dressing had been found to promote desloughing, granulation, and healing. These thereby make it suitable for the treatment of mouth sores such as aphthous ulcers [34]. In this study, whitish latex of* Carica papaya* is applied directly to the affected areas of the tooth to cure toothache and the decoction is used for treating mouth sores and oral thrush.

*Psidium guajava* is a well-known traditional medicinal plant used in various indigenous systems of medicine [35]. The leaves and bark of* P. guajava* tree have long history of medicinal uses, which is still employed today. The root is used in West Africa as a decoction to relieve diarrhea, coughs, stomach ache, dysentery, toothaches, indigestion, and constipation [35]. Stem, bark, and root-bark are astringent. The ethnomedicinal uses include the crushing of the leaves and the application of the extract on wounds, boils, skin, and soft tissue infectious site.* P. guajava* leaf is a phytotherapeutic used to treat gastrointestinal and respiratory disturbances and is used as anti-inflammatory medicine. The leaves are used in USA as an antibiotic in the form of poultice or decoction for wounds, ulcers, and toothache [36, 37]. The leaf extract possesses anticestodal, analgesics, anti-inflammatory, antimicrobial, hepatoprotective, and antioxidant activities [38–41]. The flavonoids content of aqueous extract of* P. guajava* leaves is believed to be responsible for the good antibacterial activity [42].* Psidium guajava* in this study is used to calm down toothache which may be due to analgesics, anti-inflammatory, and antimicrobial properties.

*Nicotinia tobacum* locally called tobacco or tabac in Cameroon is used for toothache by direct application of fresh leaves or ground dried leaves mixed with calcium carbonate (a mixture generally called snuff) into an infected tooth to calm down pain. Apart from its use in the management of toothache, it is also used by traditional healers from the northern regions of Cameroon in tooth whitening. Smokeless tobacco is believed to increase the circulation of endorphins which act to alter pain appreciation at different levels within the central nervous system including spinal cord, midbrain, thalamus, and cortex [43].

*Spilanthes africana* is another plant that is widely used by traditional healers in Cameroon. It is also used as a mouthwash for instant treatment of halitosis due to its peppermint taste and for the treatment of minor fractures of the teeth and alveolar bone; when applied directly to the cavity, it alleviates toothache. The use of* Spilanthes* spp. in the treatment of toothache by direct application by traditional healers had been documented in India [44]. Analgesic and anti-inflammatory activities of different* Spilanthes* spp. have made it useful for the treatment of toothache, mucositis, and sore throat and for relieving pain from boils, cut wounds, and other types of wounds in traditional medicine [44].* Spilanthes* also possess antipyretic, anticancer, antifungal, and antioxidant activities [44].

*Eucalyptus saligna* mouthwash gargle is used in Cameroon to treat mainly toothache, sore throat, and halitosis. It has been shown that the essential oil of the leaves of* Eucalyptus globulus* has antimicrobial activity against gram-negative bacteria (*E. coli*) as well as gram-positive bacteria (*S. aureus*) [45, 46] which are found in the oral cavity.

The roots of* Moringa oleifera* were also used to treat toothache in Cameroon by direct application on the tooth cavity. This plant has been found to be specific against* Staph. Aureus.*, Vibrio cholerae, and* Escherichia coli* and have no antifungal activity [47, 48]. Its antibacterial activity is responsible for its ability to calm toothache.

*Capsicum frutescens* (long pepper) have antibacterial properties against* Staph aureus* [49] and its extracts possess anti-inflammatory and analgesic effects comparable to diclofenac in experimental rat models [50].

*Ageratum conyzoides* is a herb called African panacea or the king of plants in Cameroon because it treats several diseases. It is used by traditional healers in many countries in the treatment of a wide variety of diseases. The entire parts are used for calming down pain and for tooth extractions by traditional healers. This plant possesses anticancer and antiradical properties which inhibit the growth of many microorganisms and exhibits anti-inflammatory, analgesic, and antidiarrheic properties [51, 52].

*Dichrocephala integrifolia* is an annual herb that is used by traditional healers in Cameroon for tooth extractions [53]. In in vitro studies, anticancer, antimicrobial, anti-inflammatory, and antioxidant activities of this herb have been noted [54].

*Cocos nucifera* (coconut) roots are boiled and used as mouth rinse for treating toothache and tooth sensitivity in Cameroon. Decoction obtained from coconut tree is used as mouthwash and gargle [55]. Extracts from this plant have been shown to have antibacterial, antifungal, antiviral, and antioxidant properties [55, 56]. The anticaries effect is attributed to lauric acid obtained from coconut flour, which is sensitive to* Streptococcus mutans* and as a result reduces plaque bacteria and biofilm and exerts antifungal activities [55, 56].

*Persea americana* (avocado pear) possess anti-inflammatory and antifungal properties, specific activity against* Mycobacterium tuberculosis, Streptococcus pyogenes, Staphylococcus aureus*, and varieties of fungi [57, 58]. The seeds of this plant are crushed and boiled to constitute a mouth rinse that is used in treating toothache and mouth sores by traditional healers.

*Ipomoea batatas* (sweet potato) leaves are squeezed and placed into an open cavitation of the tooth to treat toothache. Antimicrobial study has shown that low concentrations of the sweet potato freeze dried extract inhibit the growth of* Streptococcus mutans, S. mitis, Staphylococcus aureus*, and* Candida albicans* in both agar disk and agar well diffusion tests [58]. It has been shown to contain wound healing antiulcers, anti-inflammatory, antimutagenic, and antidiabetic properties [59].

*Garcinia mannii* locally called chewing stick is a tropical forest tree whose twigs are used as chewing stick or local toothbrush. Locally it has been observed to arrest dental caries or reduce long-term dental pain among people who use them daily. The chemical constituent of this plant reported in the literature is aminoflavone which requires further scientific evaluation [54].

*Garcinia kola* also known as bitter kola has been referred to as a “wonder plant” because every part of it has been found to be of medicinal importance. In Cameroon, the bark is used as a mouth rinse to stop dental pain. The roots and stems are cut into short chew sticks used for cleaning teeth.* Garcinia kola* seeds are chewed as an aphrodisiac or used to cure cough, dysentery, and chest colds, to prevent and relieve colic, and can as well be used to treat headache in herbal medicine.

*Arnica montana also known as* sun flower though used for treatment of oral infection has been shown to have slight inhibition of the adherence of the growing cells (19% for* Streptococcus mutans* and 15% for* Streptococcus sobrinus*) and of water-insoluble glucan formation (29%) at these same concentrations [60].

## 5. Conclusion

The study provides comprehensive information on therapeutic methods employed by traditional healers for the treatment of oral diseases. The identification of the active ingredients of the plants used by these traditional healers and assessment of their efficacy in the treatment may provide some useful leads for the development of new effective drugs in oral disease treatment.

## Figures and Tables

**Table 1:** Sociodemographic characteristics of the participants.

Characteristics	Male *n* (%)	Female *n* (%)	Total *n* (%)
Age (years)			
≤20	1 (0.7)	0 (0.0)	1 (0.5)
21–30	8 (5.8)	7 (11.3)	15 (7.5)
31–40	23 (16.7)	15 (24.2)	38 (19.0)
41–50	35 (25.4)	13 (21.0)	48 (24.0)
51–60	30 (21.7)	9 (14.5)	39 (19.5)
61–70	29 (21.0)	16 (25.8)	45 (22.5)
>70	12 (8.7)	2 (3.2)	14 (7.0)
Educational attainment			
Informal	23 (16.7)	9 (14.5)	32 (16.0)
Primary	51 (37.0)	13 (21.0)	64 (32.0)
Secondary	33 (23.9)	24 (38.7)	57 (28.5)
Tertiary	31 (22.5)	16 (25.8)	47 (23.5)
Residence			
Urban	82 (59.4)	28 (45.2)	110 (55.0)
Rural	56 (40.6)	34 (54.8)	90 (45.0)
Type of practices			
Herbalist	74 (53.6)	38 (61.3)	112 (56.0)
Diviners	18 (13.0)	10 (16.1)	28 (14.0)
Herbalist & diviners	46 (33.3)	14 (22.6)	60 (30.0)
Apart from your training, were you taught how to measure your medicine and how to preserve them?			
Yes	37 (26.8%)	19 (30.6%)	56 (28.0%)
No	101 (73.2%)	43 (69.4%)	144 (72.0%)
Treat disease inside or outside the mouth			
Yes	77 (56.6%)	32 (53.3%)	109 (55.6%)
No	59 (43.4%)	28 (46.7%)	87 (44.4%)
Specialist in treating mouth disease			
Yes	12 (8.7%)	6 (9.8%)	18 (9.0%)
No	126 (91.3%)	55 (90.2%)	181 (91.0)
Total	138 (69.0)	62 (31.0)	200 (100.0)

**Table 2:** Mean number of patients seen weekly by the participants.

Mean patients seen in a week			
0–4	14 (10.3)	3 (5.0)	17 (8.7)
5–8	21 (15.4)	12 (20.0)	33 (16.8)
9–12	34 (25.0)	11 (18.3)	45 (23.0)
13–16	32 (23.5)	17 (28.3)	49 (25.0)
17–20	11 (8.1)	5 (8.3)	16 (8.2)
>20	24 (17.6)	12 (20.0)	36 (18.4)

**Table 3:** Medicinal plants used for the management of oral problems.

Common names	Scientific names	Parts used	Forms of preparation	Diseases treated	Method of administration
(1)	*Acalypha *sp.	Leaves	Boil	Toothache	Gargling
(2)	*Coleus blumei*	Leaves	Paste	Sore mouth and toothache	Brushing
(3)	*Sida rhombofrica*	Whole	Chewing stick and mouth rinse	Toothache	Gargling/brushing
(4)	*Chenopodium ambrosioides*	Whole	Paste	Toothache	Placing on painful tooth
(5) Masung		Seeds	Paste	Toothache	Directing the smoke from melted paste into the mouth
(6) Atui	*Ancistrocladus abbreviatus*	Bark	Boiled bark	Toothache	Calm down pain after mouth rinse
(7) Bird eye view	*Aspilia africana*	Whole	Paste	Dry mouth and toothache	Brushing
(8) Bitter leaves	*Vernonia amygdalina*	Leaves	Solution	Toothache	Gargling
(9) Blue verbena	*Stachytarpheta* *angustifolia*	Leaves	Deconction	Toothache	Mouth rinse
(10) Castor bean	*Ricinus communis*	Leaves	Solution	Toothache	Gargling
(11) Clue de geof (clove)	*Syzygium aromaticum*	Dried fruits	Wet paste	Toothache	Pain reliever
(12) Coconut	*Cocos nucifera*	Shell and roots	Powder and whole roots	Toothache and tooth sensitivity	Direct application and boil and gaggle
(13) Cola nut tree	*Cola nitida*	Bark and fruit	Solution and paste	Sore mouth and toothache	Direct application and gargling
(14) Bitter kola	*Garcinia kola*	Bark and seeds	Mouth rinse and grinded paste	Toothache and mouth thrush	Gargling paste applied by rubbing on sore areas
(15) Dibobonji	*Alchornea cordifolia*	Stem/bark	Boil	Toothache	Mouthwash
(16) Esamba	*Pycnanthus angolensis*	Stem, bark, and leaves	Deconction	Toothache	Mouth rinse
(17) Eucalyptus tree	*Eucalyptus saligna*	Leaves	Solution/paste	Halitosis, toothache, and TMJ joint pain	Gargling
(18) Eye fowl	*Spilanthes africana (Asteraceae)*	Flower buds	Whole parts	Mouth odor, broken teeth or jaws, and toothache	Chew fresh or dry until peppery taste is felt Direct application to the cavity for toothache
(19) Garlic	*Allium sativum*	Root	Paste	Gingivitis/toothache	Direct application
(20) Guava leaves	*Psidium guajava*	Leaves	Hot mouth rinse	Toothache and mouth ulcer	Calm down pain and wound healing
(21) Long pepper	*Capsicum frutescens*	Fruits	Paste	Toothache and oral candida	Mouth rinse/gaggling
(22) Maize	*Zea maize*	Whole	Powder	Mouth lesions and bullous lesion	Brushing
(23) Mango plant	*Mangifera indica*	Bark and root/leaves	Solution	Toothache/soft tissue inflammation	Gargling
(24) Medmekube	*Acmella caurlirhiza*	Fruits	Paste	Toothache and abscess	Maceration
(25) Moringa	*Moringa oleifera*	Root	Decoction	Toothache	Mouth rinse
(26) Njansang	*Ricinodendron heudelotii*	Seeds	Paste	Toothache	Apply directly
(27) Pawpaw	*Carica papaya*	Leaf latex and leaf	Solution	Toothache, mouth sores, sore throat, and thrush	Gargling
(28) Peace plant	*Dracaena diesteliana *	Root	Solution	Toothache	Harvest fresh boil and gargle
(29) Pear tree	*Persea americana*	Seed and bark	Deconcoction	Toothache	Gaggling of hot solution
(30) Sun flower	*Arnica montana*	Leaves	Solution	Toothache	Gargling
(31) Sweet potato	*Ipomoea batatas*	Fresh leaves	Wet paste	Acute toothache/inflammation	Direct application
(32) Tobacco	*Nicotinia tobacum*	Dried or fresh leaves	Powder or wet paste	Powder for toothache and paste for tooth bleaching	Direct application of powder into cavity to arrest caries or mummify pulp tissue Direct application of fresh squeezed paste to bleach teeth

**Table 4:** Medicinal plants specific for oral diseases.

Common names	Scientific names	Parts used	Forms of preparation	Diseases treated	Method of administration
(1)	*Ocimum basilicum*	Leaves	Deconction	Sinusitis	Sniff hot vapour
(2) Bush pepper	*Piper guineense*	Fruits	Paste in water	Dental caries	Mouth rinse and direct application
(3) Orange fruits	*Citrus sinensis*	Leaves	Decoction	Gingivitis	Gargling
(4) Alakata pepper	*Aframomum danielli*	Seed	Paste	Sore mouth	Rubbing
(5) Aloe vera	*Aloe vera*	Leaves	Gel	Gingivitis	Rubbing
(6) Banana plant	*Musa cavendishii*	Roots	Solution	Sore throat	Drinkable
(7) Onion	*Allium cepa*	Leaves	Paste/deconction	Sore throat, toothache, and dental abscess	Direct application for toothache and gargling of hot fluid for sore throat
(8) Black* (bush plum)*	*Canarium* *schweinfurthii*	Dried fruits	Baked fruits in water	Tonsillitis	Gaggling
(9) Camelina	*Camelina bengalensis*	Whole	Drinkable solution	Dry mouth and thrush	Drinking
(10) Cashew	*Anacardium occidentale*	Unripe fruits	Deconction	Oral syphilis	Mouth rinse
(11) Cypress	*Cupressus bethanis*	Seed/bark	Mouth rinse solution	Bolous lesion, toothache	Gargling/paste for brushing
(12) Echinacea	*Echinacea purpurea*	Leaves/stem	Paste	Toothache, sinusitis, and oral cancer	Calm down pain on direct application
(13) Garden eggplant	*Solanum torvum*	Leaves/roots	Mouth rinse solution	Dry mouth/inflammation	Gargling/drinking
(14) Ginseng	*Lepidium meyenii*	Roots	Mouth rinse	Inflammation	Gaggling
(15) Hibiscus	*Hibiscus esculentus*	Leaves	Deconction	Sore throat	Gaggle hot liquid
(16) Chewing stick	*Garcinia mannii*	Stem	Direct chewing	Arrest caries	Chew stem

**Table 5:** Medicinal plants for dental extractions.

Common names	Scientific names	Parts used	Forms of preparation	Diseases treated	Method of administration
(1) “Native iodine”	*Arnica montana*	Leaves	Solution	Toothache/fresh wound from extraction	Rubbing (maceration)
(2) Ageratum (king plant)	*Ageratum conyzoides*	Whole	Powder	Toothache/extraction	Brushing/direct application
(3) Cotton tree	*Gossypium arboreum*	Leaves	Hot mouth rinse	Tooth extraction	Gaggling
(4) Bang-api	*Dichrocephala integrifolia*	Whole	Paste	Migraine/toothache	Massaging/tooth extraction

**Table 6:** Parts of plant used for treatment.

Part	Frequency (*n*)	Percent (%)
Leaves	49	48.1
Bark	43	42.1
Whole plant	30	29.4
Fruit	5	4.9
Seed	4	3.92
Mineral	17	16.6
Root	51	59.0
Stem	31	30.2
Total	102	100

**Table 7:** Minerals used as adjuvants with plants by traditional healers.

Minerals	Form of preparation	Diseases treated	Method of administration
White sulphur	Paste	Toothache	Direct application
Yellow sulphur	Paste	Toothache	Direct application
Calcium carbonate	Paste	Toothache	Direct application
Alum	Grounded powder	Bleeding gums and arrest of postextraction bleeding	Direct application in aqueous solution as mouth rinse
Bicarbonate solution	Aqueous solution	Mouth ulcers	Mouth rinse
